# The use of segmented regression for evaluation of an interrupted time series study involving complex intervention: the CaPSAI project experience

**DOI:** 10.1007/s10742-020-00221-9

**Published:** 2020-11-24

**Authors:** Ndema Habib, Petrus S. Steyn, Victoria Boydell, Joanna Paula Cordero, My Huong Nguyen, Soe Soe Thwin, Dela Nai, Donat Shamba, James Kiarie, the CaPSAI Project Team, Ndema Habib, Petrus S. Steyn, Victoria Boydell, Joanna Paula Cordero, My Huong Nguyen, Soe Soe Thwin, Dela Nai, Donat Shamba, James Kiarie

**Affiliations:** 1 The UNDP/UNFPA/UNICEF/WHO/World Bank Special Programme of Research, Development and Research Training in Human Reproduction (HRP Research), Geneva, Switzerland; 2 Global Health Centre, Geneva Graduate Institute, Geneva, Switzerland; 3 Population Council, Abelemkpe, Accra, Ghana; 4 Department of Health Systems, Impact Evaluation and Policy, Ifakara Health Institute, Dar es Salaam, Tanzania

**Keywords:** Complex intervention, Quasi-experiment, Interrupted time series, Segmented regression, Community-driven intervention, Modern contraception uptake

## Abstract

An interrupted time series with a parallel control group (ITS-CG) design is a powerful quasi-experimental design commonly used to evaluate the effectiveness of an intervention, on accelerating uptake of useful public health products, and can be used in the presence of regularly collected data. This paper illustrates how a segmented Poisson model that utilizes general estimating equations (GEE) can be used for the ITS-CG study design to evaluate the effectiveness of a complex social accountability intervention on the level and rate of uptake of modern contraception. The intervention was gradually rolled-out over time to targeted intervention communities in Ghana and Tanzania, with control communities receiving standard of care, as per national guidelines. Two ITS GEE segmented regression models are proposed for evaluating of the uptake. The first, a two-segmented model, fits the data collected during pre-intervention and post-intervention excluding that collected during intervention roll-out. The second, a three-segmented model, fits all data including that collected during the roll-out. A much simpler difference-in-difference (DID) GEE Poisson regression model is also illustrated. Mathematical formulation of both ITS-segmented Poisson models and that of the DID Poisson model, interpretation and significance of resulting regression parameters, and accounting for different sources of variation and lags in intervention effect are respectively discussed. Strengths and limitations of these models are highlighted. Segmented ITS modelling remains valuable for studying the effect of intervention interruptions whether gradual changes, over time, in the level or trend in uptake of public health practices are attributed by the introduced intervention.

*Trial Registration*: The Australian New Zealand Clinical Trials registry.

*Trial registration number*: ACTRN12619000378123.

*Trial Registration date*: 11-March-2019.

## Introduction

Quasi-experimental designs are widely used in public health research studies in studying causal effect especially where randomization is neither feasible nor ethical (Shadish et al. 2002; Bonell et al. 2009). An interrupted time series with a control group (ITS-CG) and a Difference-in Difference (DID) designs are two of the most commonly used quasi-experimental designs, with both incorporating a parallel control group and also permitting measuring, changes in outcomes longitudinally, before and after intervention is introduced (Lopez Bernal et al. 2018, 2019; Lopez Bernal et al. 2017; Patel et al. 2014; Rajaram et al. 2014; Serumaga et al. 2011).

In the DID design, the assessment of outcome measures usually take place at two time points; once before and once after the end of roll-out of the intervention; and it is furthermore assumed that the pre-intervention trends for the intervention and control groups are parallel. The DID design with one outcome measurement before and one after intervention introduction is also referred in literature as a simple pre-test-post-test (or a before-after) control group design (Shadish et al. 2002; Morris 2008; Zientek 2016; Lopez Bernal et al. 2019).

The ITS-CG design, on the other hand, requires multiple regular spaced outcome measurements before and after the intervention roll-out, accounts for time and allows for non-parallel trends between intervention and control groups to occur at pre-intervention (Lopez Bernal et al. 2019; Shadish et al. 2002). The ITS-CG is considered as the more powerful design than the DID (Soumerai et al. 2015; Shadish et al. 2002). The ITS design is well-suited to address some unique characteristics of the interventions being studied, including varying durations of intervention roll-out as well as delayed (lag) effects of the intervention (for its full impact to trickle) in studied population (Wagner et al. 2002; Ramsay et al. 2003). Studies utilizing longer time series with desirable minimum 100 observations have been shown to be reliable in the estimation of effect of the interventions and can robustly model the underlying trends while controlling for secular and seasonal changes as well as the random fluctuations at each time point (Shadish et al. 2002; Wagner et al. 2002). However, shorter time series designs with under ten pre- and post-intervention observations, are still being used, being comparatively more feasible, less resource-intensive than longer time series, and are used especially when the interest is in assessment of effectiveness of an intervention during limited time period. Shorter ITS-CG design is comparatively more powerful than the DID study design, for causal inference and for controlling for confounding by indication (Shadish et al. 2002; Soumerai et al. 2015).

The ITS-CG design minimizes history bias by allowing for both the before-after intervention comparison as well as the intervention-control group comparison to be made (Bernal et al. 2017; Wagner et al. 2002; Naci and Soumerai 2016). However, a control group for the ITS-CG design must be chosen carefully (Lopez Bernal et al. 2018) to ensure its baseline comparability to the group receiving the intervention, so that if an effect is detected in the intervention group but not in a well-chosen control this could be attributed to the intervention; but if the effect is detected in both the intervention and control series this would suggest presence of confounders (Lopez Bernal et al. 2018; Shadish et al. 2002). Therefore, ensuring comparability of the intervention and control groups at baseline for ITS-CG design is quite crucial and methods to achieve a covariate balance at baseline exist that include matching of factors likely to influence the primary outcome at study planning phase, as well as during analysis through use of a multivariable regression.

The ITS-CG design can also be used to evaluate the effects of the complex intervention of social accountability, defined as a combination of activities that aim to empower and educate clients to demand quality services and to support health services actors to recognize and act on citizens’ demands (Boydell et al. 2018; Steyn et al. 2019).These activities include civic and health education, group priority-setting, joint problem identification, and problem-solving that are gradually rolled out and, in turn, the effects are gradual and cumulative. At the heart of these actions are efforts to change (1) values, norms, and culture, (2) attitudes and perceptions, and (3) resources and capacities of the health service (Gullo et al. 2017). These change the behaviour of service users and providers and, in turn, contribute to improvements in service availability, access, utilization and quality of services (Boydell et al. 2018; Steyn et al. 2019). Social accountability has been established to have positive effects on health outcomes (Manandhar et al. 2004).

**Fig. 1:**
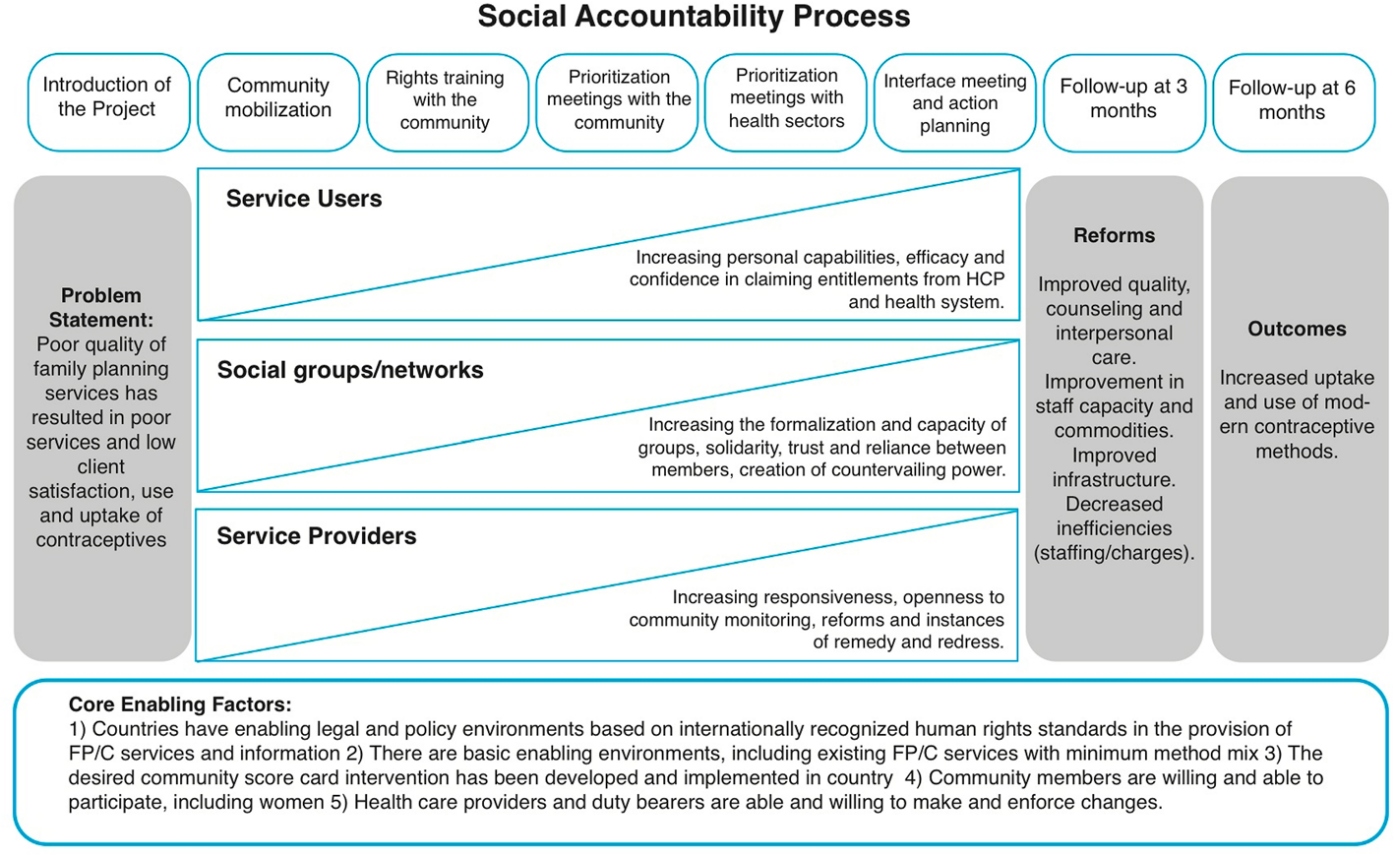
CaPSAI theory of change (HCP-health care provider)

Social accountability is a complex participatory process with multiple and interrelated components, steps and actors and several simultaneous processes of behaviour change among those delivering or receiving the intervention and adapts to local context. In line with the Medical Research Guidance on studying complex interventions (MRC 2006), the Community and Provider Social Accountability Intervention (CaPSAI) project examines both the impact and processes of a social accountability intervention in the context of contraceptive information and service provision in Ghana and Tanzania. The CapSAI theory of change processes are depicted in Fig. 1, extracted from Steyn et al. (2020), and consist of eight (8) steps/joint planned activities (top row); which involve health care service users, social groups/networks and health care service providers (HCP), working together to identify bottlenecks to accessing modern contraception; becoming self-empowered through these activities to implement the reforms needed, and which in turn are expected to lead to improvements in the uptake of modern contraception. Specifically, the combination of joint activities undertaken in these eight gradual steps consist of: (1) CaPSAI project introduction *through implementation partner meeting between local leaders and identification of stakeholders and infrastructure set-up for delivering the intervention*; (2) community mobilization *where implementation partner gathers the participants of the intervention (community members, health service providers and other duty bearers) to introduce them to the intervention*; (3) community rights training *where community is empowered by being provided with information on health awareness and education, existing service standards, rights training, good governance and accountability, who then rate the existing services against right-based standards and generate discussions about local priorities*; (4) community prioritization meetings *where implementation partner distils themes and priorities raised by the community and these groups collectively score the issues and set priority areas for action*; (5) health sectors prioritization meetings *where the implementation partner distils themes and priorities raised by service providers who then collectively score the issues and indicators and set priority areas for action*; (6) interface meeting between community and health providers and action planning *where results of prioritization meetings are presented and consensus on ranking of the priority items and the action plan is reached, and furthermore roles and responsibilities are assigned for following up to 6- to 12-month period*; (7) first follow-up meeting at 3 months *with priority areas and action items followed up with both the community and service providers; and change anticipated at this stage on the part of health services actors and that remedial actions to have taken place which should be demonstrated in the monitoring activities.* (8) second follow-up meeting at 6 months *that enables the monitoring of longer-range outcomes and on the remedy of unresolved issues raised in the first follow-up meetings*. The CAPSAI project specifically aims to measure the effect of the intervention on contraceptive uptake and use. It also aims to understand what are the mechanisms and contextual factors that influence and generate these effects.

This paper describes the application of the ITS-CG design for evaluating the effectiveness of CaPSAI on the uptake of modern contraception, and focuses on the regression modelling parameterization, estimation and interpretation of the parameter estimates arising from the segmented regression modelling. The paper also illustrates the regression modelling parameterization under the DID modelling and discusses its similarities and limitation relative to the ITS-CG segmented modelling for evaluation of the modern contraceptive uptake.

## CaPSAI project description

### Study objectives, setting and design

One of the primary objectives of the CaPSAI project is to evaluate the effectiveness of a social accountability intervention on the uptake of modern contraception, among women aged 15–49 and accessing health facilities for family planning (FP) services during an 18-month period, in Ghana and Tanzania. The protocol is registered with the Australian New Zealand Clinical Trials registry (Steyn et al. 2020).

Since the CaPSAI is being delivered at community level, it was not possible to randomly assign health facilities and corresponding catchment areas to intervention and control without risking contamination of the control group with a community level intervention. Furthermore, because of the availability modern contraception uptake data collected regularly at multiple time points in the target FP health facilities in both countries; and of a suitable parallel control group made the ITS-CG design the best suited for this study. The design involves 31 monthly assessments of the primary outcome of interest, and therefore is classified as a short time series, with 9 to 10 observations at pre-intervention and 16 to 17 post-intervention observations used for primary evaluation. The design allows used for the allocation of the intervention and control to the studied health facilities and corresponding communities to be determined in a such a way that would minimize the risk of contamination. The ITS-CG design accommodates measuring of the actual number of new users of modern contraception at monthly intervals, over several months, before, during and after the introduction of CaPSAI, that can be mapped to intervention steps and hence allow for its use to assess the timing of the effects in relation to the sequence of the intervention.

The two comparison groups consisted of a group of health facilities receiving the intervention (CaPSAI) and a group of health facilities acting as a control. The control group health facilities receive the standard of care family planning and contraception (FP/C) services as per national guidelines.

The study intervention involved community members, health professionals offering FP services at health facility level and other duty bearers within facilities’ catchment areas working together to understand their entitlements and responsibilities, and jointly identifying and implementing ways to improve the delivery and quality of FP services over a 12-month period. This process is composed of eight steps, drawn from existing social accountability projects (Boydell et al. 2018; Boydell and Keesbury 2014; Crankshaw et al. 2019), adapted to local contexts while retaining fidelity across study sites. More details regarding the composition of these steps have been outlined elsewhere (Steyn et al. 2019).

The effect (impact) of CaPSAI is anticipated to start to emerge after end of Step 6 following constructive engagement of community and health system actors (Steyn et al. 2019). While the CaPSAI is delivered at the community level and involves the health facility duty bearers and aims to affect changes in the health facility level, e.g. increased privacy or improved provider behaviour that, in turn, changes increase use of facilities and contraceptive uptake.

To ensure balance in important characteristics, as well as pretest between two groups, eight eligible facilities were selected in each the control and intervention and were frequency-matched based on geographical location (rural vs. urban/semi-urban), type of health facility (primary vs. secondary) and also on expected average number of new users per month at pre-intervention.

The catchment area of a health facility is important for assessment of health services utilization and calculation of population-based rates of outcomes of interest (Zinszer et al. 2014). Different approaches have been used to define the catchment area, among them including the distance that patients have to travel to reach the health care facility, or other methods that use some statistical measures based on case cumulative ratio (Gilmour 2010; Zinszer et al. 2014). For CaPSAI study, the catchment area is defined as the area surrounding the study facility with its residents being the primary users of the facility’s services. In Ghana, the catchment area is based on administrative area with demarcations provided by the Ghana Health Services (GHS). For Tanzania, the catchment area demarcations were provided by Ministry of Health. More description of the CaPSAI project, the intervention and study design is found in the protocol paper (Steyn et al. 2020).

### Primary outcome

The primary outcome of interest for illustration in this paper is the uptake of modern contraception, defined as the rate of new use of modern contraception, expressed as the number of women requesting new use of modern contraception, per 10,000 women of reproductive age (15–49), per month. Any woman aged 15–49 is classed as a new user of modern contraception if she requested use of modern contraception and she had (1) never before used any FP method (new acceptors); (2) switched to a modern FP method from a traditional method (additional user) *or* (3) was planning to re-start a FP method after a period of not using any method for at least 6 months (additional user) (Steyn et al. 2019).

### Data collection

A facility audit registry, based on the SARA tool, was used to retrieve aggregated monthly data on the number of new users of modern contraception in all sixteen (16) facilities in each country. The exact times of each step was recorded for each site and the timeline is split into 3 phases: 9 to 10 months of pre-intervention phase (pre-step 1), five (5) months of intervention roll-out (Steps 1 to 6) phase and 16–17 months of post-intervention phase (post-step 6). The denominator of the uptake of modern contraception, is measured at the community level, and is defined as the number of women of reproductive age 15–49 per month in the catchment area. The denominator data is captured annually through a facility audit, using GHS data in Ghana and for Tanzania Comprehensive Council Health Plan profiles calculated from the Census reports through projection or just taking 20% of the total number of women in a given catchment area was used. The denominator data is expected to remain approximately constant during the study period.

## Statistical methodologies consideration

### ITS segmented regression modelling

Segmented regression modelling is widely used for the analysis of ITS data (Lopez Bernal et al. 2017; Soumerai et al. 2015; Taljaard et al. 2014; Ansari et al. 2003; Ramsay et al. 2003; Wagner et al. 2002). Contraceptive uptake in both the intervention and control groups will be measured by comparing the level changes and trends over time during the study period using segmented regression modelling. This method involves partitioning outcome data into time intervals consistent with the times at which intervention interruptions occur and fitting a separate regression line segment to each time intervals. Intervention interruptions can occur at different points. In some cases, an intervention may be expected to show an immediate impact on the outcome. In other cases, due to gradual roll-out of an intervention, the impact may not be immediate and takes time to come into full effect, more likely after the intervention roll-out is complete. There may also be a series of interruptions introduced in the same community over time. Segmented regression modelling can account for one or more of these intervention interruptions. The regression line segments resulting from a segmented regression model can estimate the level and trend before and after one or more of these intervention interruptions have taken place.

Due to repeated outcome measurements from same facilities over time, the uptake of modern contraception will be evaluated by implementing the segmented Poisson regression and utilizing the Generalized Estimating Equations (GEE) (Diggle et al. 2002; Fitzmautice et al. 2008). An autoregressive (AR) serial covariance structure is proposed to account for serial (auto-)correlations in the GEE model, assuming observations closer in time being more correlated than those further apart, with facility as the unit of analysis. The GEE model will additionally account for correlation between health facilities, due to allocation to intervention or control group being clustered on geographical area or district. Since the CaPSAI is rolled-out gradually, the full impact of this intervention is expected to be delayed or lagged until after Step 6. Therefore, statistical analysis approaches that account for the lag must be applied to guard against the mis-specifications of the effects of the intervention (Wagner et al. 2002). In this paper we demonstrate two approaches to account for this lag in segmented ITS Poisson regression modelling. The first approach is through a two-segmented ITS Poisson regression modelling that excludes the intervention roll-out period; while the second approach use a three-segmented ITS Poisson regression modelling that includes the intervention roll-out period. Both approaches are described in detail below.

#### Approach 1: Two-segmented ITS poisson regression model that excludes the intervention roll-out period

**Fig. 2:**
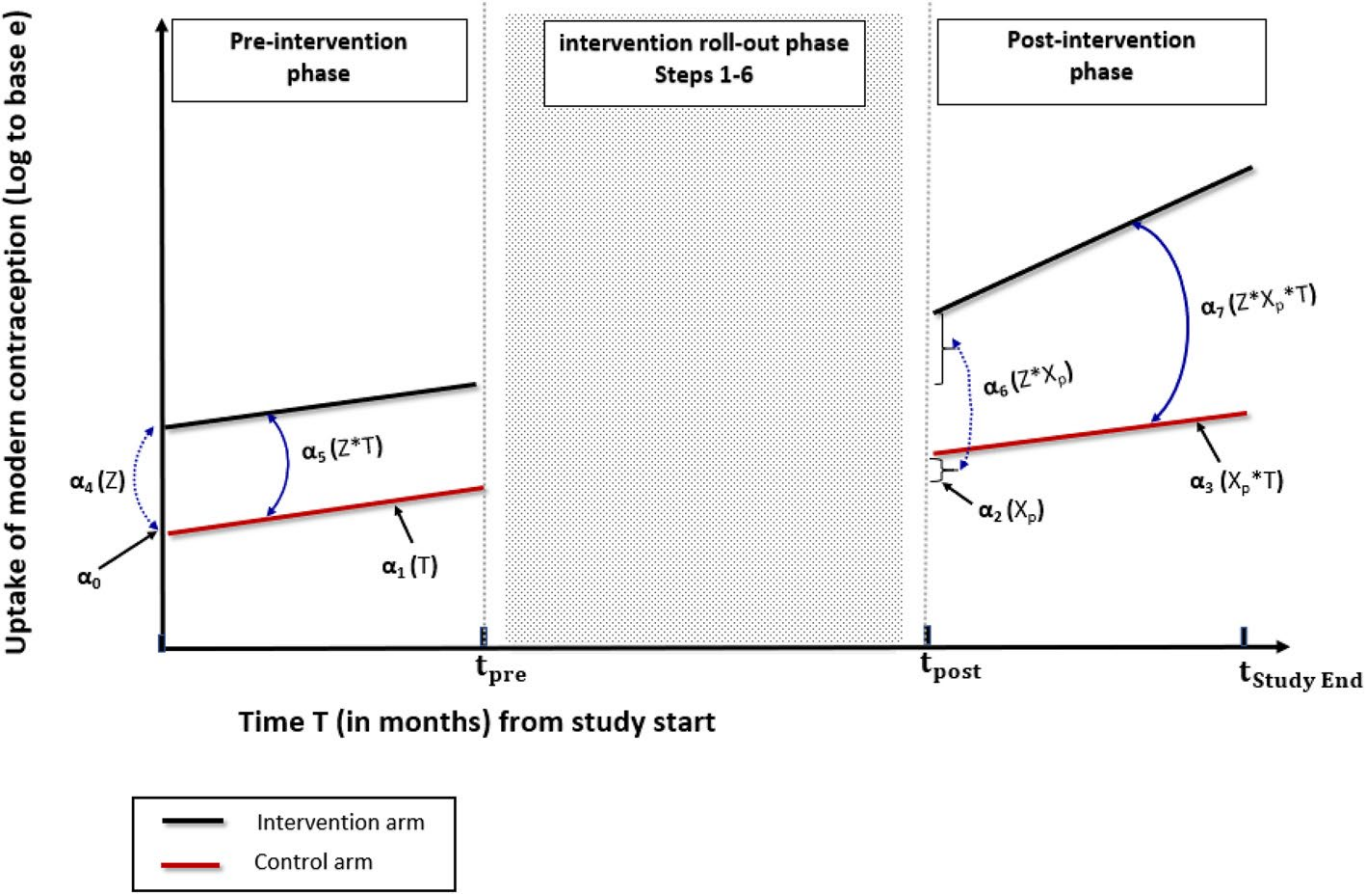
Two-segmented ITS poisson regression parameters estimates for uptake of modern contraception, for the intervention and control arms

This approach includes all the outcome data points in the analysis except those collected during the intervention roll-out (Wagner et al. 2002). In CaPSAI study context, this implies excluding the outcome data during the roll-out of Steps 1–6 (Fig. 2). Let ${t}_{pre}$ denote the pre-intervention time point (in months) just before the intervention roll-out begins and ${t}_{post}$ denote the post-intervention time point (in months) immediately following the end of intervention roll-out.

As Fig. 2 displays, the Poisson ITS GEE regression model will have two regression segments in each of the intervention and control groups: one segment for the *pre-intervention phase* covering the period from study commencement to the time ${\text{t}}_{{{\text{pre}}}}$ just prior to the start of Step 1; and the second segment covering the *post-intervention phase* period from time ${\text{t}}_{{{\text{post}}}}$ that immediately follows end of Step 6 up to the study end. The two-segmented regression model therefore compares the changes in level and in trajectory (slope or trend) in the uptake of modern contraception at the post-intervention compared to the pre-intervention period and between the intervention and control groups during same period.

The mathematical relationship between the modern contraception uptake outcome and the covariates in the two-segmented Poisson ITS regression model, is further explored. Let ${\text{Y}}_{{i,t}}$ be the monthly aggregated number of new users of modern contraception for facility unit i at month t. ${\text{Y}}_{{i,t}}$ can be expressed in terms of uptake of modern contraception ${{\uplambda }}_{i,t}$, where ${{\uplambda}} _{{i,t}}  = ({\text{Y}}_{{{\text{i}},{\text{t}}}} /{\text{catchment}}\;{\text{population}}\;{\text{size}}\;{\text{for}}\;{\text{facility}}\;i)$. This regression model can hence be expressed in terms of ${\text{log}}\left( {\uplambda _{{i,t}} } \right)$, as follows:

$$   {\text{Model}}\;{\text{1:}}\;{\text{Log}}(\lambda _{{i,t}} ) = \alpha _{0}  + \alpha _{1} {\text{T  +  }}\alpha _{2} {\text{X}}_{{\text{p}}} {\text{  +  }}\alpha _{3} {\text{TX}}_{{\text{p}}}  + \alpha _{4} Z + \alpha _{5} Z{\text{T  +  }}\alpha _{6} Z{\text{X}}_{{\text{p}}}  + \alpha _{7} Z{\text{X}}_{{\text{p}}} {\text{T  +  }}\epsilon _{{i,t}},    $$

where $\lambda _{{i,t}}  = {\text{uptake}}\;{\text{of}}\;{\text{modern}}\;{\text{contraception}}\;{\text{use}},\;{\text{for}}\;{\text{facility}}\;{\text{unit}}\;i,\;{\text{at}}\;{\text{month}}\;{\text{t}}$, ${\text{T}} = {\text{time}}\left( {{\text{months}}} \right)\;{\text{rom}}\;{\text{study}}\;{\text{start}}$ (with integer $\text{T}$: $0 \le {\text{T}} \le {\text{t}}_{{{\text{pre}}}}$; ${\text{t}}_{{{\text{post}}}}  \le T \le {\text{t}}_{{{\text{Study}}\;{\text{End}}}}$),

$$ Z = {\text{Intervention}}\;{\text{group}} = \left\{ {\begin{array}{*{20}l}    0 \hfill & {{\text{if}}} \hfill & {{\text{Control}}\;{\text{health}}\;{\text{facility}}} \hfill  \\    1 \hfill & {{\text{if}}} \hfill & {{\text{Intervention}}\;\left( {{\text{CaPSAI}}} \right)\;{\text{health}}\;{\text{facility}}} \hfill  \\   \end{array} } \right.   $$

$$  {\text{X}}_{{\text{p}}}  = {\text{Post-intervention}}\;{\text{phase}} = \left\{ {\begin{array}{*{20}l}    0 \hfill & {{\text{if}}} \hfill & {{\text{T}} \le {\text{t}}_{{{\text{pre}}}} } \hfill  \\    1 \hfill & {{\text{if}}} \hfill & {T \ge {\text{t}}_{{{\text{post}}}} } \hfill  \\   \end{array} } \right.    $$

$$  {\text{X}}_{{\text{p}}}  = {\text{Post-intervention}}\;{\text{phase}} = \left\{ {\begin{array}{*{20}l}    0 \hfill & {{\text{if}}} \hfill & {{\text{T}} \le {\text{t}}_{{{\text{pre}}}} } \hfill  \\    1 \hfill & {{\text{if}}} \hfill & {T \ge {\text{t}}_{{{\text{post}}}} } \hfill  \\   \end{array} } \right.    $$, $\epsilon _{{i,t}}  = \rho \epsilon _{{i,t - 1}}  + u_{{i,t}} ,{\text{is}}\;{\text{a}}\;{\text{random}}\;{\text{error}}\;{\text{term}}\;{\text{for}}\;{\text{the}}\;\;{\text{modern}}\;{\text{contraception}}\;{\text{uptake}}\;{\text{outcome}}\;{\text{for}}\;{\text{facility}}\;i$, following $1{\text{st}}\;{\text{order}}\;{\text{autoregressive}}\;\left( {{\text{AR}}1} \right)\;{\text{process}},$ with an autocorrelation parameter $\rho$ equal to the correlation coefficient between adjacent error terms, such that $\left|\rho \right|<1$, and the disturbances $u_{{i,t}}  \sim N\left( {0,\sigma ^{2} } \right)$.

$T{X}_{p}$ is a two-way interaction term between the phase of intervention (${\text{X}}_{\text{p}}$) and time ($T$); and $ZT$ a two-way interaction between the allocation group ($Z$) and time ($T$), respectively. The term $Z{X}_{p}T$ is a three-way interaction between allocation group ($Z$), phase of the intervention (${X}_{p}$) and time ($T$). The uptake of modern contraception parameter (expressed as *log to base e*) at pre- and post-intervention segments is denoted as ${{\upalpha }}_{\text{i}}$ (with integer $i$ ranging $0\le i\le 7$).

Consider the *pre-intervention segment* of the model in Fig. 2. Parameter ${{\upalpha }}_{0}$ is a pre-intervention intercept for the control group, that represents the level of the uptake of modern contraception at study commencement; and ${{\upalpha }}_{1}$ a pre-intervention slope representing the modern contraception uptake trajectory, also for the control group. Parameters ${{\upalpha }}_{4}$ and ${{\upalpha }}_{5}$ represent respective differences in the uptake levels and uptake trajectories in modern contraception, for the intervention group relative to the control at pre-intervention.

For the *post-intervention segment* of the model (Fig. 2), parameter ${{\upalpha }}_{2}$ represents the change in uptake level in the control group, at the point where the intervention is fully implemented (immediate post-intervention) and ${{\upalpha }}_{3}$ is an estimate of change in uptake trajectory during the post-intervention phase; while parameters ${{\upalpha }}_{6}$ and ${{\upalpha }}_{7}$ represent the respective differences between the intervention and control groups, in the uptake levels at immediate post-intervention and in the post-intervention trajectories of uptake modern contraception. Parameters ${{\upalpha }}_{4}$ and ${{\upalpha }}_{5}$ are important in establishing whether the intervention and control groups are balanced on both the uptake level and trajectory at the pre-intervention period, which is needed to assure that any differences in uptake of modern contraception occurring during the post-intervention period are attributable to the introduced intervention. The statistical significance of either or both of parameters ${{\upalpha }}_{6}$ and ${{\upalpha }}_{7}$ and especially when the pre-intervention phase is well balanced between the two groups, would be an indication of the significant intervention effect.

#### Approach 2: Three-segmented ITS poisson regression model that includes the intervention roll-out period

**Fig. 3:**
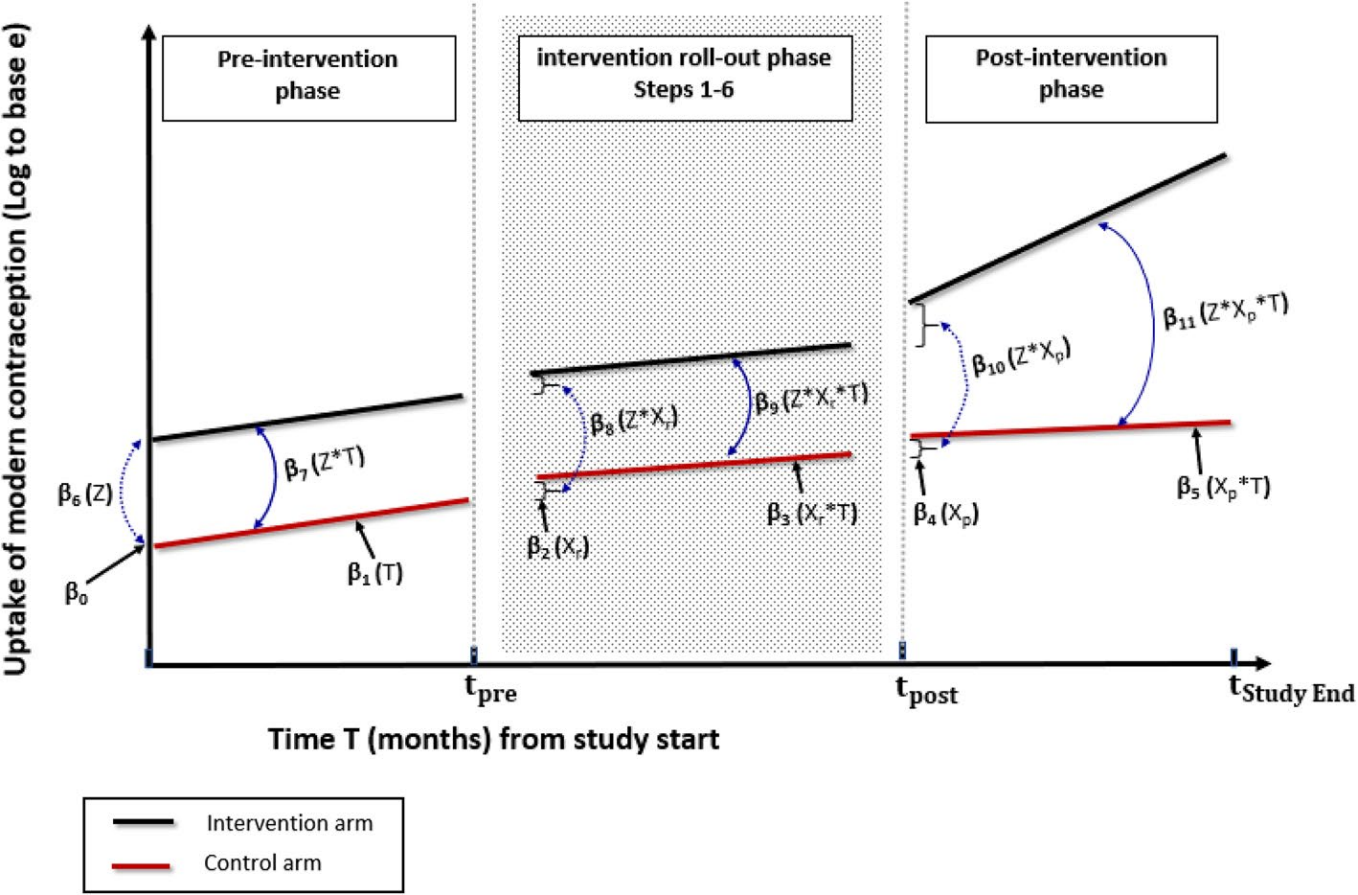
Three-segmented ITS Poisson regression parameters estimates for uptake of modern contraception, for the intervention and control arms

The three-segmented ITS model, allows for the period during the intervention *roll-out* to be modelled as a separate segment in addition to the *pre-intervention* and the *post-intervention* segments (Wagner et al. 2002; Taljaard et al. 2014). The three segments introduced therefore account for any changes in the outcome associated with the intervention during intervention *roll-out* and the *post-intervention* period (Fig. 3).

The three-segmented regression model can compare the level and trajectory of the uptake in modern contraception in all the three segments. The three-segmented regression model for log of uptake, ${{\uplambda }}_{t}$, will take the following form:

$$   {\text{Model}}\;{\text{ 2:}}\;Log(\lambda _{{i,t}} ) = \beta _{0}  + \beta _{1} T + \beta _{2} X_{r}  + \beta _{3} TX_{r}  + \beta _{4} {\text{X}}_{{{\text{p*}}}}  + \beta _{5} T{\text{X}}_{{{\text{p*}}}}  + \beta _{6} Z + \beta _{7} ZT + \beta _{8} ZX_{r}  + \beta _{9} ZX_{r} T + \beta _{{10}} Z{\text{X}}_{{{\text{p*}}}}  + \beta _{{11}} Z{\text{X}}_{{{\text{p*}}}} T + \epsilon _{{i,t}}      $$

with ${{\uplambda }}_{i,t}$,$\text{T}$, $Z$ and $\epsilon _{{i,t}}$ as previously defined; and additionally,

$$ {\text{X}}_{{\text{r}}}  = {\text{Intervention}}\;{\text{roll-out}}\;{\text{phase}}\;{\text{or}}\;{\text{later}} = \left\{ {\begin{array}{*{20}l}    0 \hfill & {{\text{if}}} \hfill & {{\text{T}} \le {\text{t}}_{{{\text{pre}}}} } \hfill & {} \hfill  \\    1 \hfill & {{\text{if}}} \hfill & {T > {\text{t}}_{{{\text{pre}}}} } \hfill & {} \hfill  \\   \end{array} } \right. $$

$$ {\text{X}}_{{{\text{p*}}}}  = {\text{Post-intervention}}\;{\text{phase}} = \left\{ {\begin{array}{*{20}l}    0 \hfill & {{\text{if}}\quad {\text{T}} < {\text{t}}_{{{\text{post}}}} } \hfill  \\    1 \hfill & {{\text{if}}\quad T \ge {\text{t}}_{{{\text{post}}}} } \hfill  \\   \end{array} } \right. $$

The uptake parameters (in *log to base e*) of new modern contraception use at pre-intervention, intervention roll-out and post-intervention segments are denoted as ${{\upbeta }}_{\text{j}}$ (with integer $j$ ranging $0\le j\le 11$). As can be noted, the covariate assignment value for ${\text{X}}_{\text{p}{*}}$ in model 2 is not the same as covariate assignment for ${\text{X}}_{\text{p}}$ in Model 1. For Model 2, ${\text{X}}_{\text{p}{*}}$ equals to 0 at any time from study commencement to the end of implementation strategy roll-out (i.e. value is zero in the pre-intervention and roll-out phases); while for Model 1, ${\text{X}}_{\text{p}}$ equals to 0 from study start to just prior to commencement of the implementation strategy roll-out (i.e. value is zero only in the pre-intervention phase, and is not applicable in roll-out phase as this phase is excluded from the analysis).

Consider the *pre-intervention segment* of the model (Fig. 3). Parameters β_0_ and β_1_ represent the level of the uptake of modern contraception at the start of the study (intercept) and its trajectory (slope) respectively, for the control group; while β_6_ and β_7_ represents the respective differences in the uptake level and trajectory for the intervention group relative to the control prior to the roll-out of the intervention. The parameters β_6_ and β_7_ will establish whether the intervention and control groups are balanced in uptake level and trend during the pre-intervention period.

For the *intervention roll-out segment* of the model (Fig. 3), parameters β_2_ and β_3_ for the control group provides the estimates of the change (relative to the start of pre-intervention phase) in uptake of modern contraception level at the point of beginning of the intervention roll-out (CaPSAI Step 1) and its trajectory from that point to the point where intervention roll-out is complete. Parameters β_8_ and β_9_ represent the differences between intervention and control in the uptake levels at the start of the roll-out and in the trajectory of the uptake to the end of the roll-out phase, respectively. If parameters β_8_ or β_9_ or both exhibit statistical significance in favour of intervention group, it would imply an early impact of the CaPSAI a as it is being rolled-out.

Figure 3 furthermore displays the *post-intervention segment* of the ITS model, whereby parameters β_4_ and β_5_ respectively represent the uptake level immediately following intervention roll-out completion (immediate post-intervention) and uptake trajectory from that point to the end of study for the control group; while parameters β_10_ and β_11_ respectively represent the difference in the uptake levels at immediate post-intervention and in the uptake trajectories at post-intervention, between the intervention and control groups. If β_8_ and β_9_ are not significant, while either or both of β_10_ and β_11_ are significant in favour of intervention group, and provided similar distribution of parameters at pre-intervention, this would imply a lagged but significant impact of the CaPSAI on the uptake of modern contraception.

### DID regression modelling

**Fig. 4:**
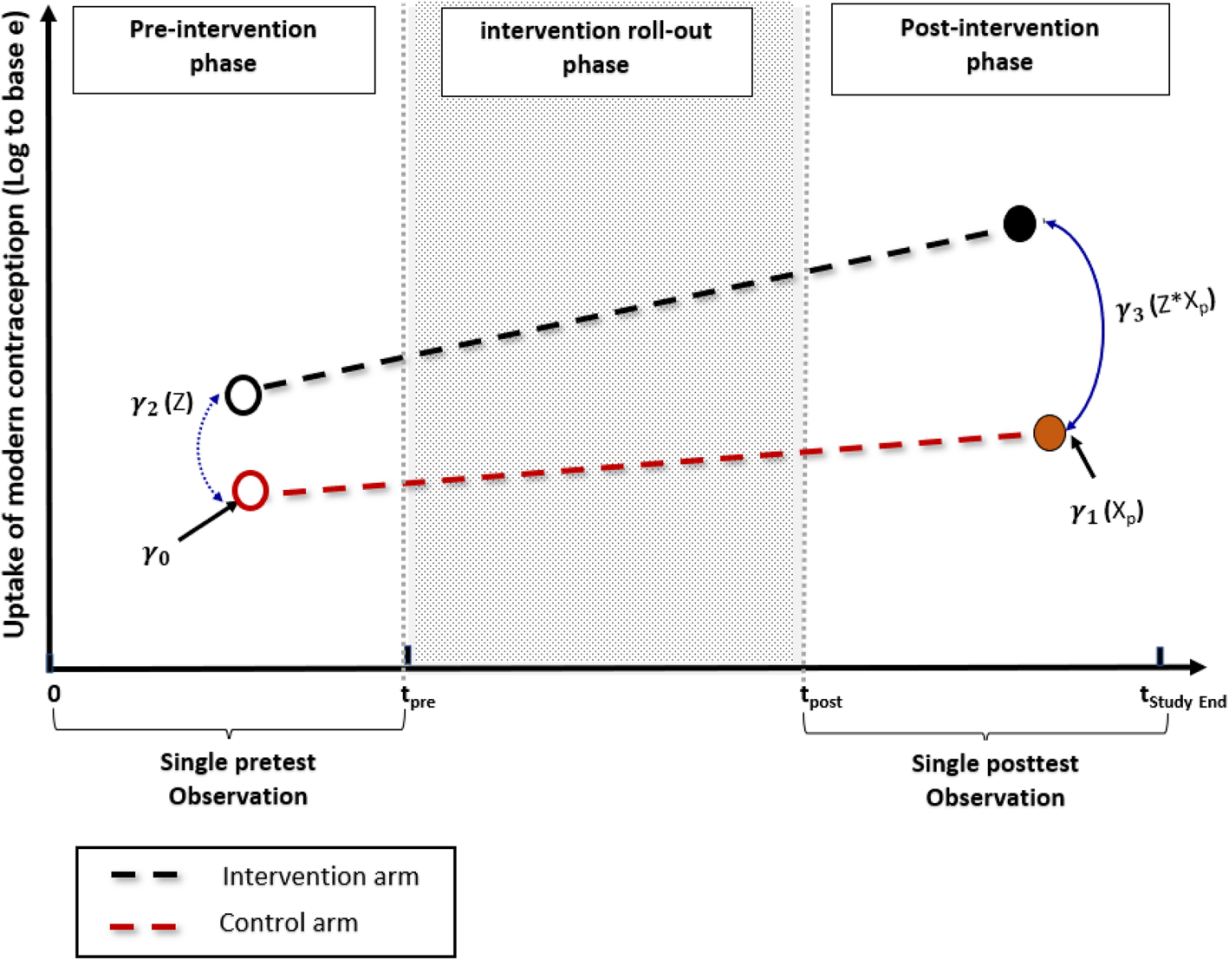
DID Poisson regression parameter estimates for uptake of modern contraception, for the intervention and control arms

To facilitate comparison with the segmented regression modelling under the ITS-CG design, we have included the Poisson Difference-In-Difference GEE regression model parameterization under the current CaPSAI study design (Fig. 4). The model is formulated as follows:

$$  {\text{Model}}\;{\text{3:}}\;\log (\lambda _{{i,t}} ) = \gamma _{0}  + \gamma _{1} {\text{X}}_{P}  + \gamma _{2} Z + \gamma _{3} Z{\text{X}}_{P}  + \epsilon _{{i,t}} ; $$

where the modern contraception uptake during time period t, is given by $\uplambda _{{i,t}}  = \left( {\overline{{Y_{{i,t}} }} /{\text{catchment}}\;{\text{population}}\;{\text{for}}\;{\text{facility}}\;i} \right)$; with t representing two time periods; at pre-intervention ($0\le {T\le t}_{pre})$ and at post-intervention (${\text{t}}_{{{\text{post}}}}  \le T \le {\text{t}}_{{{\text{StudyEnd}}}}$); whereby $\overline{{Y_{{i,t}} }}  = \frac{1}{n}\sum\nolimits_{{j = 1}}^{{j = n}} {Y_{{i,j}} }$ is the number of new users averaged over n months, for facility i, in each of the two time periods; and ${Y}_{i,j}$, the observed number of new users in the ith facility during jth month (j = 1,2…,n).

$$ Z = {\text{Intervention}}\;{\text{group}} = \left\{ {\begin{array}{*{20}l}    0 \hfill & {{\text{if}}} \hfill & {{\text{Control}}\;{\text{health}}\;{\text{facility}}} \hfill  \\    1 \hfill & {{\text{if}}} \hfill & {{\text{Intervention}}\;\left( {{\text{CaPSAI}}} \right)\;{\text{health}}\;{\text{facility}}} \hfill  \\   \end{array} } \right. $$

$$ {\text{X}}_{p}  = {\text{Post-intervention}}\;{\text{phase}} = \left\{ {\begin{array}{*{20}c}    0 & {{\text{if}}} & {T \le t_{{pre}} }  \\    1 & {{\text{if}}} & {T \ge {\text{t}}_{{{\text{post}}}} }  \\   \end{array} } \right. $$

$Z{X}_{p}$ is a two-way interaction term between the allocation group ($Z$) and phase of intervention (${\text{X}}_{\text{p}}$). $\epsilon _{{it}}  = {\text{random}}\;{\text{error}}\;{\text{term}}\;{\text{for}}\;{\text{the}}\;{\text{uptake}}\;{\text{measure}}\;{\text{for}}\;\;{\text{facility}}\;i,\;{\text{following}}\;1{\text{st}}\;{\text{order}}\;{\text{autoregressive}}\;\left( {{\text{AR}}1} \right)\;{\text{process}}$ $= \rho \epsilon _{{i,t - 1}}  + u_{{i,t}}$, with autocorrelation parameter $\rho$ equal to the correlation coefficient between error terms in the two *pre*- and *post*-intervention time periods such that $\left|\rho \right|<1$, and the disturbances $u_{t}  \sim N\left( {0,\sigma ^{2} } \right)$.

The uptake of modern contraception parameter (expressed as *log to base e*) at pre- and post-intervention segments is denoted as ${{\upgamma }}_{\text{i}}$ (with integer $i$ ranging $0\le i\le 3$).

Consider the *pre-intervention phase* as shown in Fig. 4. Parameter ${{\upgamma }}_{0}$ is an intercept for the control group, that represents the level of the uptake of modern contraception (monthly average) at the pre-intervention period; and ${{\upgamma }}_{2}$ represents the excess level of uptake in modern contraception, in the CaPSAI group relative to the control, during this period.

For the *post-intervention phase* (Fig. 4), parameter ${{\upgamma }}_{1}$ represents a change in the level of uptake in the control group; while ${{\upgamma }}_{3}$ represents an excess change in uptake level in the intervention group relative to the control, after full implementation of the intervention. The statistical significance of parameter ${{\upgamma }}_{3}$, would be an indication of the significant CaPSAI effect on change in the level of uptake of modern contraception. The DID regression model parameterization, hence shows DID models usefulness in allowing for testing for statistically significance of the resulting DID parameter estimates.

## Discussion

As illustrated through the mathematical formulation, both DID and ITS-CG segmented regression models allow for comparing intervention and control groups before and after intervention introduction and therefore testing of the statistical significance of the uptake parameters.

Controlling for biases is very important for the internal validity of research findings. Relevant to this study, possible biases include history bias and contextual confounding bias. For CaPSAI study, history bias can occur when non-study interventions or other activities affecting modern contraception uptake, are implemented during the same period as the roll-out of the study intervention. The non-study interventions could be for example, FP awareness campaigns, policy or political changes that could affect community or provider behaviour towards contraception and contraceptives use. Because both DID and ITS models incorporate a control group, the history bias is hence minimized. The potential bias due to contextual confounding was addressed at design stage through matching of the control and intervention facilities based on geographical location, facility type and average number of new modern contraception users at pre-intervention. The contextual confounding can also be addressed in analysis using the multivariable DID and ITS-CG regression models.

There are limitations to using the DID models relative to the segmented ITS-CG models for the modern contraception uptake evaluation. The first limitation is that assumption of parallel trend for the DID regression model, is not always demonstrable and therefore confounding due to group differences is an issue (Lopez Bernal et al. 2019). For the ITS-CG segmented models, trends are allowed to differ between the intervention and control groups, with resulting differences between them at pre-intervention accounted for during modelling, and as illustrated with more parameters being estimated under the segmented ITS-CG models than in DID models. The DID models, for example, cannot provide the estimates of the trend in uptake of modern contraception, which is another limitation of this model, especially when significant trends exist.

Using segmented ITS regression models that accommodate for non-linear trends, adds flexibility to modelling of outcomes, especially when they are non-linear over time. For this study, we opted for a linear trend for both segmented models presented due to the CaPSAI study design being a short time series (with short duration of observation and with under 100 data points).

The ITS-CG design has its own challenges, one of which is its application in the evaluation of complex interventions consisting of several components being introduced at different times (gradual roll-out) (Taljaard et al. 2014). In this case adding several interruptions in the ITS regression model reflecting the times at which these different components are introduced in the studied community would address this issue. The first six sequentially rolled-out steps of the CaPSAI study (Fig. 1) were sub-grouped into those related raising awareness (Steps 1–3); and those related to constructive engagement of priorities in FP (Steps 4–6). These steps 1–6 are all crucial for the evaluation of impact of CaPSAI on the modern contraception uptake and are considered as a single package of intervention and therefore will be evaluated as one interruption (CaPSAI roll-out phase). The gradual roll-out of the CaPSAI is expected to lead to a time lag in intervention effect being experienced, covering the period between the CaPSAI initial introduction (Step 1) to when full effects would be observed (after end of Step 6).

As has been demonstrated in this paper, both types of segmented regression models can adequately account for this time lag, by either excluding the lag period or adding the lag as an additional interruption in the ITS model. The two-segmented regression model accounts for the lag by removing from analysis the period of the intervention roll-out (Steps 1–6) and therefore only evaluates the modern contraception uptake levels and trajectories between pre- and post-intervention periods in each group, as well as the excess uptake level and deviation in trend in the post-intervention period between the intervention and control groups. The three-segmented regression accounts for the time lag by modelling the intervention roll-out segment as the third segment in addition to the pre- and post-intervention segments and has an additional advantage (over two-segmented regression model) assessing if there is an early impact of the intervention on uptake even during the roll-out of the intervention. In case of a statistically significance in change in level and/or trajectory parameter estimates at post-intervention in favour of the intervention group, while levels and trajectories are similar at pre-intervention period, this will be a demonstration of effectiveness of the CaPSAI on increasing modern contraception uptake. On the other hand, the DID method cannot model this lag (intervention roll-out) period (Fig. 4) and must exclude this period from the analysis.

The advantage of the three-segmented over the two-segmented model is its ability to model the data during the lag-period (intervention roll-out phase) and therefore use all the data collected over the entire study period, with the ability to elucidate any early effects of the intervention. For the CaPSAI study, the duration of roll-out is expected to last about five (5) months, which enables evaluation of level and trend in uptake during this phase. The current study design limitation is its inability to assess the contribution of each of the 6 components/steps of CaPSAI on the level and uptake of modern contraception during the intervention roll-out phase, in terms of estimation of parameter estimates between these, because of limited number of data points to do this between these steps. It is because of this reason that for the CaPSAI study, we can only estimate the intervention effect level and trend parameters only at the start of roll-out (step 1) i.e. excess level ${\beta }_{8}$; and after the end of step 6, i.e. excess trend ${\beta }_{9}$ respectively (Fig. 3). Therefore, we can, using the three-segmented model, only detect if there was an intervention effect during the roll-out phase but without identifying the step at which it started to be significant.

However, there are limitations that segmented methodologies on their own cannot be able to address. One of the limitations, is the models’ inability to account for intervention coverage deficiencies in the communities in question. The performance of the models (in terms of statistically significant results) is fully dependent on whether the intervention is successfully rolled-out to all the facilities and respective catchment communities as intended; the absence of which have the potential to dilute the intervention effect towards the null effect. The second related limitation is dose-response oriented, specifically, the degree of completeness of the implementation of the reforms deemed necessary by the community, by the end of step 6. The degree of completeness and hence quality of reforms implemented by the end of intervention roll-out (which also depends on the intensity of reforms needed as well as other logistics), may have an influence on community satisfaction and in turn on how they respond to modern contraception uptake, by the time of post-intervention assessment (Step 7 onwards). The models cannot account for this dose-response scenario, nor account for variation in satisfaction levels between communities receiving the intervention, whose effect can go either way.

Due to repeated nature of the outcome data per facility over time in CaPSAI study, dependency of observations at facility level is expected and must be accounted for. The use of marginal ITS model applying general estimating equations (GEE) is an accepted way to account for clustering or repeated longitudinal observations (Hanley et al. 2003). GEE allows for correcting for autocorrelation of repeatedly collected outcomes within facilities and in turn guard against underestimation of the outcome standard errors of the time-dependent predictors(Ansari et al. 2003), in this case exposure to intervention (or control). The marginal GEE regression model is therefore selected as the most suitable for the CaPSAI study since the primary interest is to estimate the population averaged parameters of uptake in modern contraception.

When autocorrelation is considered in the model, the probability of falsely concluding significant intervention effect when there is none (false Type I error) is minimized. The choice of the correlation structure for the CaPSAI study is based on the study design. It is expected that the facility level data collected at time points closer in time to be likely to be highly autocorrelated than those that are further apart. The dependency structure following the first order autoregressive process AR(1) is hence proposed. Higher order autoregressive structures may be considered if they result in a better model. Since the GEE modelling is based on quasi-likelihood estimation, the adequacy of the model utilizing AR(1) will be compared to other higher order dependency structures using the Quasi Information Criteria (QIC); with the best model leading to the lowest QIC value. The plot of the partial autocorrelation function (PACF) against its lagged values is among ways that will be used towards exploring the appropriateness of the AR(1) compared to AR dependencies of higher orders.

Another type of correlation that could be present in the time series data is the seasonal autocorrelation, however this will not be accounted for in the model for CaPSAI study since it requires least 24 monthly data points, distributed on the same calendar month at pre- and post-intervention(Wagner et al. 2002).

The accurate identification of the health facility catchment area is crucial for understanding the population served, for planning and evaluation of service delivery that include access to services, and erroneous estimation of catchment area can lead to potentially flawed results (Zinszer et al. 2014). For CaPSAI study, this error if present, could lead to inaccurate estimation (under- or over-estimation) of the uptake of contraception for both control and intervention facilities. However, because the denominator of the rate is assumed to remain constant throughout the study and what changes over time is the numerator of the rate, this is not expected to affect the estimation of the changes in uptake in modern contraception. The potential for inaccuracies in the catchment area estimation the CaPSAI study were additionally minimized by using data from national sources.

Compared to other types of quasi-experimental designs including the DID designs, the interrupted time series design with a parallel control group has the best statistical performance when used to evaluate the effect of complex interventions on improving health outcomes, through use of routine data collected from a homogeneous sample of units or clusters, which would allow for baseline comparability between the intervention and control groups. As is the case for the ITS-CG design, half of the clusters are assigned to receive the intervention while the remaining half serve as control (do not receive the intervention), with this considered ethical if there is no prior indication of benefit of the intervention on the outcome being studied. Studies that have evaluated the effectiveness of a social accountability intervention on the first-time use (or uptake) of the modern contraception are limited, especially in the context of repeatedly measuring of the outcome over time. However, the effect of social accountability on current use of modern FP methods has been previously studied which utilized a simple pre-test post-test cluster randomized trial (Gullo et al. 2017). The following conditions have to be met in order to use the ITS-CG design and to be able to apply the segmented regression modeling: (1) presence of routine data collection; (2) ability to roll-out intervention to the relevant clusters during the same period; (3) known start date, for those intervention that roll-out quickly, or both the start and end dates, for the interventions that take time to roll-out; (4) sufficient number of data points or observations to enable estimation of levels and trends, in the pre-intervention, during intervention roll-out and post-intervention phases, as needed, that would allow the use of the segmented ITS regression modelling approach outlined; and (5) an adequate control group.

We therefore conclude that an interrupted time series study with a parallel control group must be well-designed to adequately address ethical, logistical and internal validity concerns; and that the segmented regression modelling approach that accounts for important sources of variation, is recommended in the evaluation of whether the introduced study intervention is responsible for changes in the level and/or trend in uptake of useful public health practices.

## Data Availability

No data is associated with this article.
